# Creating a virtual, clinical scenario based teaching programme for foundation doctors in Leeds

**DOI:** 10.1192/bjo.2021.394

**Published:** 2021-06-18

**Authors:** David Hall, Thomas Lane, Alexander Harbinson

**Affiliations:** Leeds and York Partnership NHS Foundation Trust

## Abstract

**Aims:**

Through consultation with foundation doctors on their psychiatry placements in Leeds, we established that they didn't feel the trustwide teaching programme met their needs, with them rating the relevance as 5.8 on a 1-10 Likert scale. They also reported their access to formal and informal teaching had been limited by COVID-19 restrictions. We aimed to create an accessible teaching programme that met their developmental needs as set out by the Foundation curriculum, as well as their confidence and skill set in dealing with common mental health presentations. Our supplementary aims were to promote psychiatry as a career and to provide supervised teaching opportunities to core trainees.

**Method:**

Having assessed the foundation doctors confidence in dealing with different scenarios, we created a 9 week teaching programme covering common mental health presentations they're likely to encounter during their training. The virtual sessions focus on what assessment and management skills would be expected for a foundation doctor, as well as when and how to access support and refer on. The programme was designed to be trainee led with the teaching being facilitated by core trainees as it was felt that they would best relate to the experiences and developmental needs of foundation doctors. This also provided the CTs with an opportunity to develop their teaching skills, something that has become more difficult during COVID.

**Result:**

Through weekly feedback of the sessions we were able to demonstrate that for 8 of the 9 sessions the foundation doctors rated them as being ‘useful’ or ‘very useful’ and we're currently reviewing the topic and materials for the outlying session.

Through self-assessed ratings before and after the programme, we demonstrated significant increases in confidence in dealing with all 9 of the scenarios. All of the foundation doctors indicated that they had found the programme beneficial.

**Conclusion:**

As shown in the results, the programme has been well received by the foundation doctors who's confidence in dealing with a range of scenarios has improved. The programme has also been well received by the trust who have asked us to repeat the it for future foundation rotations and by core trainees who were grateful of the development opportunities that this provided. Further developments will include extending the programme for the duration of the placement to enable us to cover an enhanced range of presentations and to consolidate core skills.

